# 

**Published:** 1895-08

**Authors:** 


					﻿Vol. XV.
AUGUST, 1895.
NO. 8.
THE
HOMEOPATHIC pHY^lClAfl,
A Monthly Journal of Homoeopathic Materia Medica
and Clinical Medicine.
" He is a freeman whom truth makes free, and all are slaves beside."
"Seek the Truth: come whence it may, cost what it well.”
BDITBD AND PUBLISHED BY
WALTER M. JAMES, M. D.
PHILADELPHIA:
2STO. 1231 LOCUST STREET.
LONDONI
ALFRED HEATH & CO., Sole Agents for Great Britain,
114 Ebury Street, Eaton Square.
49“ Fearly Subscription, 99.SO, invariably in advance. Single numbers, 96 etc.
Intend at the Philadelphia Poet-Offlce at Second-Claae Mail Matter.
				

